# Cutting-edge technology and automation in the pathology laboratory

**DOI:** 10.1007/s00428-023-03637-z

**Published:** 2023-11-06

**Authors:** Enrico Munari, Aldo Scarpa, Luca Cima, Matteo Pozzi, Fabio Pagni, Francesco Vasuri, Stefano Marletta, Angelo Paolo Dei Tos, Albino Eccher

**Affiliations:** 1 Pathology Unit, Department of Molecular and Translational Medicine, University of Brescia, Piazza Del Mercato, 15, 25121 Brescia, BS Italy; 2 Pathology Unit, Department of Diagnostics and Public Health, University of Verona, Verona, Italy; 3 Pathology Unit, Department of Laboratory Medicine, Santa Chiara Hospital, APSS, Trento, Italy; 4 Bruno Kessler Foundation, Trento, Italy; 5 University of Trento, CIBIO Department, Trento, Italy; 6 Pathology Unit, Department of Medicine and Surgery, University of Milano-Bicocca, IRCCS Fondazione San Gerardo Dei Tintori, Monza, Italy; 7 Pathology Unit, IRCCS, Azienda Ospedaliero-Universitaria Di Bologna, Bologna, Italy; 8 Department of Pathology, Pederzoli Hospital, Peschiera del Garda, Italy; 9 Surgical Pathology and Cytopathology Unit, Department of Medicine-DIMED, University of Padua School of Medicine, Padua, Italy; 10 Section of Pathology, Department of Medical and Surgical Sciences for Children and Adults, University of Modena and Reggio Emilia, University Hospital of Modena, Modena, Italy

**Keywords:** Automation, Pathology, Innovation, Quality, Efficiency

## Abstract

One of the goals of pathology is to standardize laboratory practices to increase the precision and effectiveness of diagnostic testing, which will ultimately enhance patient care and results. Standardization is crucial in the domains of tissue processing, analysis, and reporting. To enhance diagnostic testing, innovative technologies are also being created and put into use. Furthermore, although problems like algorithm training and data privacy issues still need to be resolved, digital pathology and artificial intelligence are emerging in a structured manner. Overall, for the field of pathology to advance and for patient care to be improved, standard laboratory practices and innovative technologies must be adopted. In this paper, we describe the state-of-the-art of automation in pathology laboratories in order to lead technological progress and evolution. By anticipating laboratory needs and demands, the aim is to inspire innovation tools and processes as positively transformative support for operators, organizations, and patients.

## Introduction

Efforts to standardize surgical pathology laboratory processes and reduce manual work have increased over the past decades, aiming to enhance diagnostic accuracy and patient care outcomes. The handling of anatomic pathology samples is critical, as loss or incorrect storage can have serious diagnostic, legal, and ethical implications. Recommended conditions for storage include controlled temperature and humidity for paraffin-embedded blocks and secure, traceable systems for glass slides [1, 2]. The Italian Ministry of Health’s Superior Health Council has highlighted these issues in their guidelines [3].

From collection to storage, it is crucial to maintain a secure and controlled chain of custody for biological samples, ensuring quality, traceability, and proper conservation. Improving compliance and process efficiency requires solutions that automate and simplify labelling, archiving, and search processes. Automation, defined as the use of devices to replace or supplement human effort in a process [4], is key.

Standardization in tissue processing, analysis, and reporting [5] is a major focus in surgical pathology, ensuring precision and repeatability of diagnostic findings, as well as clarity in diagnostic reporting. New technologies, including digital pathology systems and artificial intelligence techniques, are being developed and applied to enhance diagnostic accuracy, though adoption has been gradual due to concerns about data privacy, cost, and compatibility [6, 7]. Also, molecular pathology provides results that need to be precise and require standardized analytical procedures before implementation [8]. In this setting, quality assurance and control systems play a crucial role, serving as adjuvants to ensure the accuracy and reliability of results [9–11].

Proper tracking, storage, and conservation of specimens are critical, impacting diagnostic accuracy, patient care, and research. The evolution of surgical pathology and patient care relies heavily on the adoption of advanced technologies and standardized practices. This paper describes the state-of-the-art in pathology laboratory automation, aiming to inspire innovation tools and processes to support operators, organizations, and, most importantly, patients.

## Pre-analytical processes

The pre-analytical phase of tissue processing comprises all the steps, starting from receiving tissue specimens to the submission of histopathology slides for interpretation [1].

**Fig. 1:**
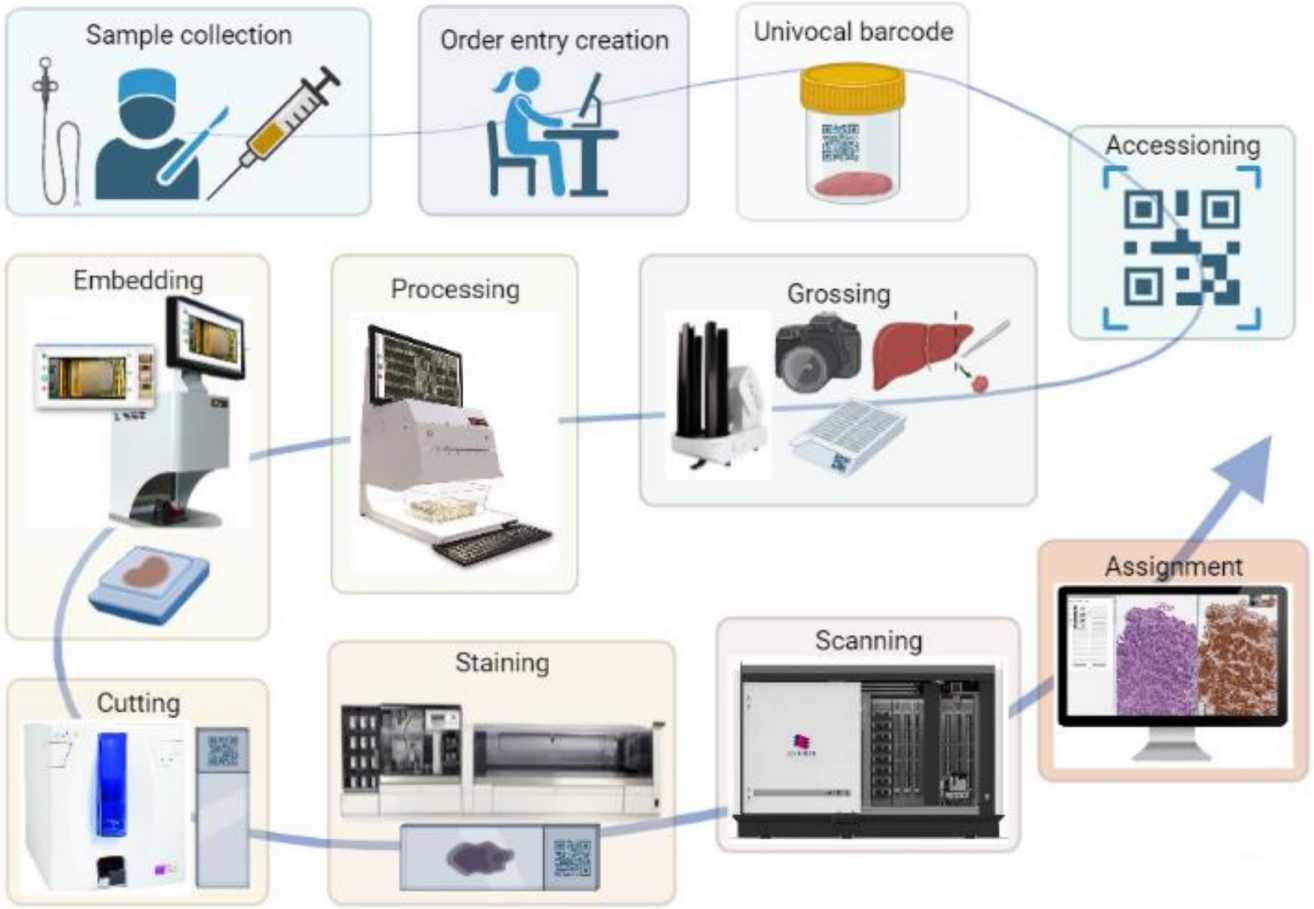
Automated workflow in the pre-analytical processes

The application fields of automation in the pre-analytical phase of pathology include specimen collection and tracking, processing, embedding, cutting, and staining (Fig. 1 and Table 1).

**Table 1:** Benefits of automation in the pre-analytical phase

Tracking samples	Tissue processing	Tissue embedding
Error reduction Faster turnaround time Control over the specimen’s progress Barcode scanning & LIMS for traceable location/status	Increased uniformity Reduced discrepancy Simplified quality control Enhanced efficiency	Improved productivity Increased standardization Increased reliability Less manual labor Less variability
*PathTracker™ (SPOT Imaging, Sterling Heights, USA);* *FinderFLEX (LOGIBIOTECH, Italy)*	*Tissue-Tek Xpress®* × *120 tissue processor (Sakura Finetek, Japan); HistoCore PEGASUS Plus tissue processor (Leica Biosystems, Germany)*	*Tissue-Tek AutoTEC® a120 (Sakura Finetek, Japan); Synergy system (Milestone, Italy)*
Microtome	Slide staining and coverslipping	Collaborative robots
Improved consistency Improved accuracy Faster turnaround time Constant thickness	Optimized workflow Greater reproducibility Improved precision Increased reliability	Improved workflow Less human labor Reduced errors Reduced contamination risks
*AS-410M (Dainippon Seiki, Japan); Tissue-Tek AutoSection® (Sakura Finetek, Japan)*	*VENTANA BenchMark (Roche Diagnostics, Switzerland); BOND systems (Leica Biosystems; Germany); Tissue-Tek Genie® system (Sakura Finetek, Japan)*	*Tissue-Tek SmartConnect® (Sakura Finetek, Japan)*

Examples of products available on the market are presented in italics

### Tracking samples

It has become vital to create automated systems that can assist with tracking and managing the workflow of the specimens due to the rising volume of specimens received. In order to track the location, status, and stage of processing for each specimen, barcode scanning equipment and laboratory information management software (LIMS) are utilized [12, 13].

Labs can decrease the possibility of human mistakes and accelerate the turnaround time for diagnostic tests by automating the workflow process for specimens. Automated systems, for instance, can generate barcode labels that can be applied to the specimen container for tracking and notify laboratory employees when a specimen is received. The specimen’s location and status in the LIMS can be updated by scanning the barcode as it passes through each stage of processing. This enables the laboratory staff to keep track of each specimen’s progress, see any delays or problems that require attention, and know who did a certain action with the specimen and when.

PathTracker™ (SPOT Imaging, Sterling Heights, MI, USA) is a laboratory solution for bulk barcode scanning that incorporates technology to acquire, process, analyze, and log all the barcodes in the field of view, with a reported scanning time of 30 s for a 150 cassette processing basket. Any damaged or poorly printed barcodes are flagged, and PathTracker™ then provides a set of correction tools to correct damaged barcodes automatically or manually, thus ensuring continuous workflow.

FinderFLEX (LOGIBIOTECH, Alseno, Italy) is a robotic unit for handling and scanning cytohistological samples. Thanks to a multi-articulated mechanical arm, FinderFLEX can handle and insert slides, macrosection slides, biopsy cassettes, super mega cassettes, and vials into the appropriate racks in a fully automated and secure way. The operator simply has to turn on the device and log in to add any new samples for storage. Using a latest-generation barcode scanner, FinderFLEX also rapidly scans any barcodes, QR codes, and Data Matrix 2D codes, communicating directly with the LIS to ensure a systematic and traceable sample management and handling process. FinderFLEX identifies and manages the samples directly from their standard racks and containers, significantly reducing handling times, also thanks to the automatic transmission of the gripper fingers. The device is also equipped with simple and intuitive software and a touchscreen panel, which the operator can access in total safety in case of an emergency.

### Tissue processing

The importance of standardization in tissue processing within anatomic pathology cannot be overstated. It ensures uniformity by reducing discrepancies and confirming that any variations are due to the samples themselves. This uniform approach simplifies quality control, making it easier to detect and rectify any issue, like the presence of contaminants [14]. Moreover, it enhances the precision of diagnostic tests by preventing changes in tissue structure or composition that could influence subsequent analyses. Finally, it optimizes lab workflow, enhancing efficiency and saving resources.

In the past, pathologists and technicians would spend countless hours manually preparing tissue samples for diagnosis. Tissue fixation and processing may now be carried out rapidly, precisely, and with a minimum of human involvement because of the development of automated methods and tools.

The Tissue-Tek Xpress® × 120 tissue processor (Sakura Finetek, Tokyo, Japan) allows continuous streamlining of the histology workflow using vacuum infiltration to offer consistent results in rapid time, distributing cases uniformly and decreasing workloads, processing large tissue specimens in 2.5 h.

Running numerous protocols simultaneously on a single instrument is made possible by the HistoCore PEGASUS Plus tissue processor (Leica Biosystems, Wetzlar, Germany), providing a completely integrated system with the capacity to record each cassette individually, including cassette ID, amount, and color, as well as basket ID, user ID, and reagent information.

Compared to manual processing, automated tissue fixation and processing have a number of benefits. First, since all processes are meticulously regulated by the computer, automation lessens the possibility of mistakes and unpredictability in tissue processing. This may result in more precise and reliable diagnostic findings, enhancing patient care and outcomes. The second benefit of automation is quicker processing times since the computer can regulate the timing of each step to maximize effectiveness. Finally, automation frees up laboratory staff to concentrate on important duties like quality control.

### Automation in tissue embedding

One of the most critical steps in the histology procedure is embedding; after the tissue processing stage, this laborious operation is done manually and requires proper training and experience. The correct orientation of the tissue within the paraffin is of paramount importance since a badly oriented specimen will result in an uninformative section and can lead to tissue loss at cutting, with detrimental consequences for the patient. The technician embeds surgical specimens and biopsies one at a time, making sure they are positioned correctly, which is frequently a laborious and time-consuming process. For this operation to produce the best circumstances for the cutting phase, trained specialists with good manual dexterity are needed.

Compared to manual embedding, automated embedding systems have a number of benefits, such as improved productivity, standardized processing, and less manual labor.

By automating the process of embedding tissues as part of the processing protocols, the Synergy system (Milestone Medical, Sorisole, Italy) eliminates the need to manually reopen the cassettes and reposition the tissues. A carefully created rack, specialized molds, and pads make up the Synergy technology system. Through the use of a single tissue processing and embedding methodology, the sponges used for the pads guarantee the specimens’ correct orientation and facilitate cutting at the microtome stage.

The Tissue-Tek AutoTEC® a120, in conjunction with Tissue-Tek® Paraform® cassettes and Tissue-Tek® Paraform® Tissue Orientation Gels (Sakura Finetek, Tokyo, Japan), is a component of Sakura’s SMART automation concept to automate the manual work and produce a continuous flow in the lab. Such gels are made to securely hold and keep tiny tissue samples oriented. A complete system for automating cassette embedding with a throughput of up to 120 cassettes per hour is provided by the Tissue-Tek® Paraform® Sectionable Cassette System once the tissue is correctly oriented at grossing. This system locks the specimen during processing and embedding, minimizing tissue loss and eliminating the need for specimen reorientation.

These types of automated embedding systems appear to be superior compared to manual embedding, especially in terms of productivity and uniformity. Automated embedding can increase the accuracy and reliability of diagnostic testing by lowering the possibility of human error and variability. It must be kept in mind, however, that tissues can vary greatly in size, shape, and consistency, and not all may be suitable for automatic embedding. Some delicate or irregularly shaped samples may require manual embedding to ensure proper orientation and preservation.

### Automatic microtome

Microtomes, the cornerstone of pathology labs since the nineteenth century, have radically transformed tissue analysis by producing ultra-thin sections for detailed cellular structure examination and disease pathology investigation. Despite their indispensable role, microtome operation remains an artisanal task, demanding skillful handling and precise adjustments. The critical challenges of section thickness variation and tissue distortion call for innovative approaches and advanced automation to ensure reliable, reproducible results. The automated microtome operates by slicing the tissue sample into thin slices with the help of a motorized cutting blade. The instrument’s control panel allows for the generation of tissue sections with various thicknesses by adjusting the section’s thickness. The instrument’s automation also makes sure that tissue segment thickness is constant, lowering the possibility of mistakes and inaccurate diagnostic findings.

In this regard, an automatic microtome AS-410M has been developed by Dainippon Seiki (Nagaokakyo, Japan), which automatically performs high-precision and quality histological cuts according to the pre-established requirements for each case or tissue. The cut is then transferred to a slide where it is deposited and stretched; subsequently, the slide is stored in a drying chamber from where it can be collected. The cuts obtained are very homogeneous and of high quality. In addition, the equipment may include roughing modules, cut quality control, slide printing, and connection to the Laboratory Information System (LIS) for full traceability of the samples. The approximate production is 250 blocks in a 7-h work shift, with the possibility to run 24 h a day.

Sakura’s Tissue-Tek AutoSection® Automated Microtome offers one-touch trimming and customizable sectioning, coupled with numerous integrated safety measures. It aligns the block with the blade edge, ensuring precise XYZ positioning. This system enables consistent block orientation, regardless of prior trimming or sectioning on other microtomes, thus conserving both tissue and technician efforts.

Some limitations in applying such technology could be related to the fact that extremely hard or brittle materials might be challenging to cut consistently; moreover, very little biopsy might still require human hands and expertise to avoid the loss of precious tissue.

### Automation in slide staining and coverslipping

The adoption of automated staining technology has accelerated the processing of huge sample numbers while also minimizing human error, enhancing consistency, and improving staining procedures’ efficiency and dependability.

Hematoxylin and eosin (H&E)-stained slides represent the cornerstone of morphological diagnosis, and their importance cannot be overestimated [15]. Every step of the process can be automated, resulting in greater reproducibility, precision, and reliability; notably, it has been shown that automated individual staining protocol(s) as opposed to batch-stained slides might be preferable for digital pathology [16]. An example of an individual slide staining system is the Ventana HE 600 (Roche Diagnostics, Basel, Switzerland).

Using labeled antibodies, immunohistochemistry (IHC) is a potent diagnostic method in pathology that enables the identification of particular antigens in tissue slices. IHC staining has become more efficient thanks to automation, which has optimized incubation periods, temperature ranges, and reagent concentrations—elements crucial for precise antigen–antibody reactions. The automated systems also reduce background noise and non-specific staining, which raises the signal-to-noise ratio and the overall caliber of the stained slides.

Modern automated staining systems have been created by different companies to meet the various needs of pathology labs.

One example is the VENTANA BenchMark line of automated slide stainers (Roche Diagnostics, Basel, Switzerland), which provides complete IHC and in situ hybridization (ISH) staining solutions.

The BOND-PRIME automatic staining platform from Leica Biosystems (Wetzlar, Germany) can adapt to different workflow demands like batch, continuous, single slide, or STAT cases, or a combination of these for both IHC and ISH. Another example is the Tissue-Tek Genie® system from Sakura Finetek (Tokyo, Japan), a fully automated, random access stainer for IHC and ISH, with independent staining stations for handling slides with different antibodies and probes simultaneously and at any time.

A key step in the preparation of a high-quality histological glass slide is coverslipping. The quality of the coverslipping is important since the presence of air bubbles, excess or lack of mounting medium, and dried mounted slides can impair the diagnosis. There are three types of coverslipping methods, namely, the classic glass coverslip, the liquid method, and the film method. The film method is the only automatic and has been demonstrated to be the fastest, with significantly less air bubbles and staining alterations compared with the other two methods [17], thus resulting in the best method for the production of glass slides for digital scanners.

### Collaborative robots

In numerous situations, it is challenging to automate manual processes. Devices, even those manufactured by the same company, often lack sufficient coordination to transfer materials. A common daily laboratory task involves moving sections between rack systems, such as transferring samples from a staining platform to a coverslipping device [18]. The process can be time-consuming and may result in material loss due to the risk of components falling or breaking.

There is ample opportunity for enhancing production flow and intelligently integrating various steps, in addition to the need for further process development. The increasing adoption of robotic systems for material transfer across processes is a product of collaborative robotics. Collaborative robots, or “cobots,” feature sensors that facilitate safe human–robot interaction without necessitating protective barriers. Flexible, camera-assisted gripping devices also contribute to the functionality of these systems, allowing them to operate effectively. The Tissue-Tek SmartConnect® from Sakura (Tokyo, Japan) represents a cutting-edge technological advancement in laboratory automation, bridging the gap between human expertise and efficient, reliable processes. This collaborative robot has been designed to work seamlessly alongside laboratory technicians, assisting in various tasks while promoting accuracy and productivity. Once the Tissue-Tek Xpress® × 120 is loaded through SmartConnect, automated tissue processing begins. SmartConnect then independently transfers the magazines to the Tissue-Tek AutoTEC® a120 embedder. Ultimately, SmartConnect delivers standardized, high-quality, embedded blocks prepared for microtomy. Laboratories can therefore improve workflow, lower human error, and boost overall effectiveness by putting such a system in place.

Additionally, the incorporation of cutting-edge technology into these systems, such as machine learning and artificial intelligence, might result in even more precise and accurate treatment of samples, enhancing the overall outcomes.

Such robots can benefit the lab by.

- eliminating manual and accidental errors as well as contamination risks;
- simplifying routine activities and improving processes and workflows;
- increasing productivity and efficiency;
- ensuring complete sample tracking and traceability, guaranteeing their quality;
- reducing repetitive manual processes performed by health care staff, freeing up more time for strategic activities with high added value;
- helping improve patient satisfaction and, most importantly, patient safety.

## Analytical processes

**Fig. 2:**
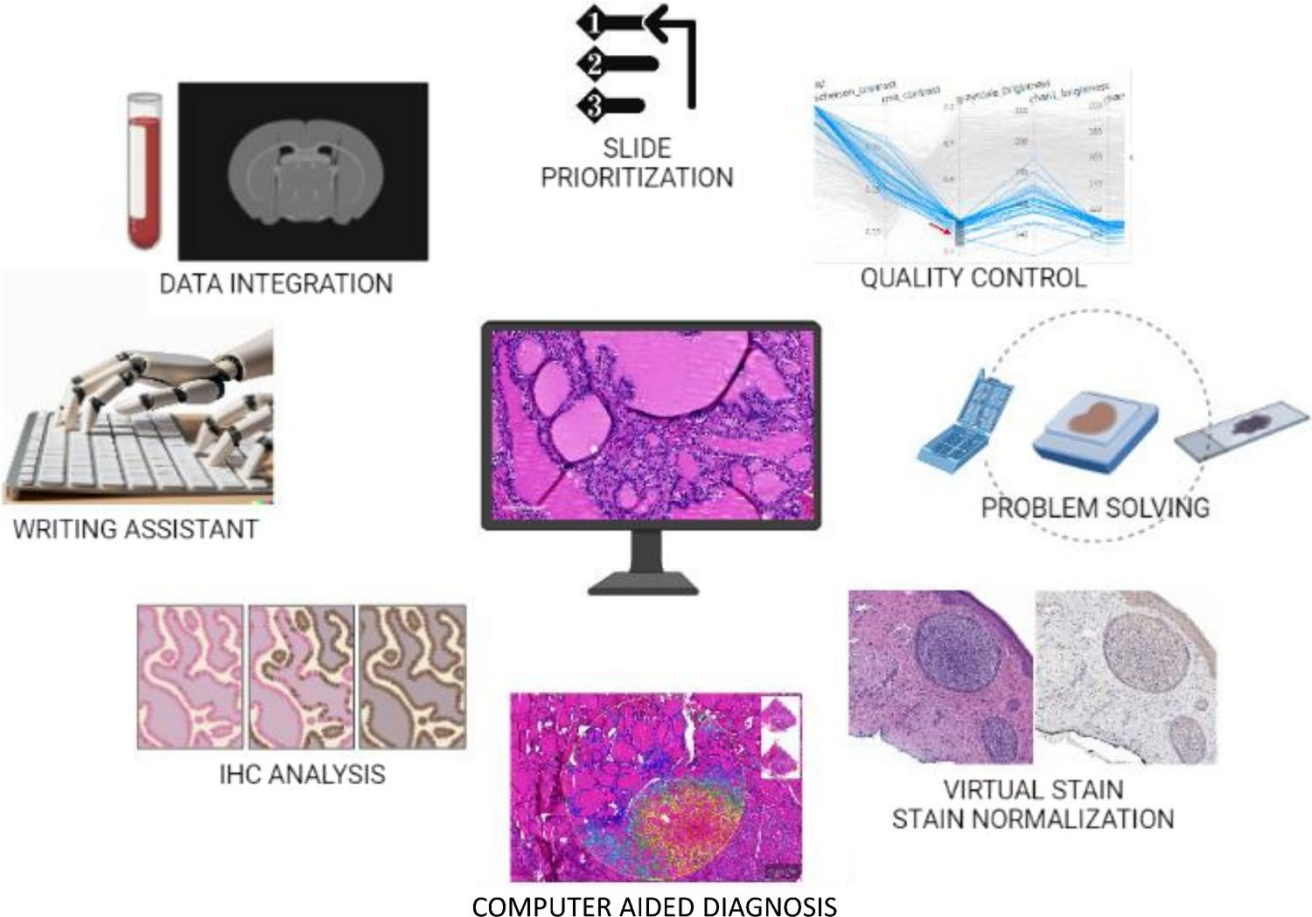
Automated workflow in the analytical processes

The application fields of automation in the analytical phase of pathology include digital pathology and the analytical process performed by computational pathology algorithms (Fig. 2 and Table 2), as well as synoptic reporting and data entry templates.

**Table 2:** Benefits of automation in the analytical phase

Digital and computational pathology	Synoptic reporting and data entry templates
Digital pathology allows remote collaboration, diagnostic precision, and efficiency Digital pathology transforms education and learning process AI-driven computational pathology improves diagnosis with advanced algorithms AI-driven computational pathology predicts treatment response from WSIs Challenges: privacy, standardization, and validation	Foster uniformity and comprehensiveness Checklists offer consistent diagnostic data and save time Checklists benefit care quality and research Standardization amplifies clarity, communication, and information exchange between institutions

### Digital and computational pathology

Modern diagnostic medicine’s crucial domains of digital pathology and computational pathology are redefining how doctors approach the analysis, diagnosis, and treatment of diseases [7].

Pathology glass slide scanners have revolutionized digital pathology by enabling the conversion of histological samples on glass slides into high-resolution digital images. This has enhanced information accessibility, storage, and sharing, fostering global collaboration among health care professionals. Over time, these scanners have significantly improved in terms of speed, resolution, and capabilities.

Notable products in the market include the NanoZoomer series (Hamamatsu, Hamamatsu City, Japan), Aperio (Leica Biosystems, Wetzlar, Germany), IntelliSite (Philips, Eindhoven, the Netherlands), Pannoramic series (3DHISTECH, Budapest, Hungary), and Axioscan (Zeiss, Oberkochen, Germany). Each of these devices offers impressive image quality, processing speed, and capacity, catering to the diverse needs of diagnostic laboratories and research facilities.

Pathologists may see, examine, and exchange high-resolution digital images of histological and cytological samples thanks to the digitalization of glass slides using whole slide imaging, or “digital pathology.”

Through the use of this technology, pathologists may now collaborate remotely, consult with specialists around the world, improve diagnostic precision, and speed up patient care [19].

The development and implementation of machine learning algorithms and artificial intelligence (AI) to evaluate digital slides is the subject of computational pathology. Using this method, quantifiable data may be extracted from the digitized slides and utilized to discover unique patterns and biomarkers, increasing diagnostic accuracy and enabling tailored medication [6].

Numerous developments in whole slide imaging technologies, rising processing power, and the accessibility of enormous annotated datasets have all contributed to the growth of digital and computational pathology. Investment and research in these areas have also increased as a result of the growing need for diagnostic solutions that are more effective, accurate, and cost-efficient.

The diagnosis of cancer is one of the main uses of digital and computational pathology. When it comes to the automated diagnosis and categorization of different tumor forms, including breast, lung, and prostate cancer, machine learning algorithms have achieved extraordinary results. These algorithms can examine digital histopathology images to find neoplastic cells, differentiate benign tumors from malignant ones, and even identify the subtypes and grades of the malignancies [20–23].

In this regard, such technology may reduce the workload for pathologists, reduce interobserver variability, and enable more reliable and precise diagnoses by automating these procedures [24].

In order to pinpoint potential beneficial uses of AI in pathology, Heinz et al. conducted an anonymous online survey involving 75 domain experts in computational pathology from both academic and industrial backgrounds [25]. The survey results suggested that the most promising future application is seen as predicting treatment response directly from standard pathology slides.

As a matter of fact, among different applications in translational medicine, digital pathology is being actively investigated to predict response and identify patients most likely to respond to treatment.

In the era of immune-oncology, the selection of patients who may benefit the most from immune checkpoint inhibitor-based therapies (ICI) like PD-1/PD-L1 blockade is a major and still unsolved issue [26]. Notably, besides PD-L1 expression on tumor and immune cells, the immune contexture represented by tumor-infiltrating lymphocytes (TILs) has been demonstrated to have strong predictive potential [27].

Interestingly, Park et al. have developed an AI-based algorithm for the analysis and quantification of TILs in the tumor microenvironment, capable of defining three immune phenotypes (IP): inflamed, immune-excluded, and immune-desert [28]. These authors demonstrated that patients with inflamed tumors have a better prognosis both in terms of OS and PFS and, in particular, that patients with inflamed neoplasms and high expression of PD-L1 show a significant improvement in survival compared to patients with high expression of PD-L1 but non-inflamed tumors.

Such results underscore the fact that the application of image analysis offers increased accuracy and efficiency by automatically measuring multiple parameters that are impossible to achieve by eye.

Besides tumor pathology, computational pathology is also being investigated and applied in critical but often neglected fields like transplantation pathology, a highly specialized area of pathology that examines both post-transplant graft biopsy results for rejection or graft damage as well as organ donor biopsy for organ allocation, as well as in many different fields of functional and non-neoplastic pathology [29–31].

Overall, it appears clear that digital and computational pathology provide very useful methods for managing and interpreting massive datasets from various sources, such as genomics, proteomics, and clinical data, in the age of big data. Through the use of machine learning algorithms, integrative data analysis, a greater understanding of disease causes, and the discovery of novel possibilities for diagnosis, prognosis, and treatment are all made possible.

One of the primary obstacles to the integration of digital pathology into clinical practice, as perceived by administrators, is associated with its cost. In this regard, Ho and colleagues elaborated a financial projection for digital pathology implementation at a large health care organization in order to estimate potential operational cost savings [32]. The projected savings were based on two main benefits associated with the use of digital pathology: (1) potential improvements in workflow/productivity and lab consolidation; and (2) avoided treatment costs due to reduced rates of interpretive errors by general, non-subspecialist pathologists. The authors projected that the total cost savings over 5 years could reach approximately $18 million. This suggests that if the costs of acquiring and implementing digital pathology do not exceed this value, the return on investment becomes attractive to hospital administrators.

Currently, different integrated digital pathology systems are being implemented around the world, providing clear examples of the feasibility of the implementation of digital pathology workflows both in small and large pathology departments supporting large and distributed health care organizations with complex patient demographic profiles [33–35]; also, official guidelines have been published [36].

Finally, the value of digital pathology in the education of anatomic pathology is beyond doubt, with a growing number of resources like digital pathology atlases. Essential skills like identifying features, providing differential diagnoses, annotating, taking photographs, describing, and presenting are all improved through the use of such resources. The way these resources are used seems to play a crucial role in overcoming the reluctance to use digital tools among certain learners. Regularly integrating these resources into unidentified case discussions, educational collections, and tutorials has the potential to dramatically improve and speed up the learning process [37].

Despite significant advancements and prospective applications, digital and computational pathology still has a number of issues that need to be resolved. Because medical data is sensitive and professionals must share photos and information, privacy and security issues with the data are raised. To ensure consistency and interoperability across various systems and organizations, it is also crucial to standardize digital imaging processes, data formats, and annotation strategies. Machine learning algorithms must also undergo thorough validation and testing before being integrated into clinical processes in order to guarantee their dependability and clinical utility [38]; strategies for preventing model accuracy losses in the contest of artifacts must also be developed [39].

We believe that the new generation of pathologists, besides having solid and comprehensive anatomic pathology training, will also need to expand their cultural background to include at least the basic principles of computational pathology and image analysis in order to bridge the cultural gap between medicine, computer science, and data analytics. This will not mean that pathologists will have to be a sort of hybrid professional, but surely they will need to have the ability to collaborate with computer scientists to understand and overcome the possible limitations of new technological approaches and, importantly, to be the main actors in this paradigm shift.

The future of digital and computational pathology is still bright, despite these difficulties, as it will make it possible to significantly increase diagnosis accuracy and give patients a more thorough grasp of disease processes by combining new imaging modalities with machine learning algorithms.

### Structured synoptic reporting

The practice of structured reporting in pathology is of utmost significance as it fosters uniformity and comprehensiveness in recording vital cancer data. This standardization not only amplifies the clarity and usefulness of reports for immediate patient care but also guarantees that invaluable data is systematically captured for secondary purposes such as research, quality assurance, and public health management.

The International Collaboration on Cancer Reporting (ICCR) has played a pivotal role in propelling this global standardization, thereby contributing to enhanced patient outcomes and breakthroughs in cancer research [40, 41].

The ICCR envisions improving patient outcomes through internationally standardized pathology reporting. The formulation of evidence-based datasets, encompassing all significant and current reporting information for any specific cancer, results in more exhaustive pathology cancer reports, refined cancer staging, and the optimization of treatment protocols for cancer patients.

Beyond the development of datasets, the ICCR has pinpointed two additional key areas of focus for the future. The first is the translation of datasets into multiple languages to expedite the adoption of reporting standards in both developed and low-to-middle-income countries (LMICs). The second is the conversion of dataset standards into machine-readable formats to facilitate their electronic implementation and global data interoperability.

The creation of evidence-based datasets, which include all essential and contemporary reporting details for each specific cancer, not only leads to more thorough pathology reports on cancer but also improves cancer staging and fine-tune treatment approaches for cancer patients [42]. Furthermore, these datasets represent the basis for the creation of nationwide networks between pathology laboratories as is the case with the Pathological Anatomy National Automated Archive (PALGA) that has been operating in the Netherlands since 1971. The aim of such organization is to promote communication and information exchange between participating laboratories and to provide potentially useful data for health care professionals in the interest of patient care and research [43].

## Post-analytical processes

**Fig. 3:**
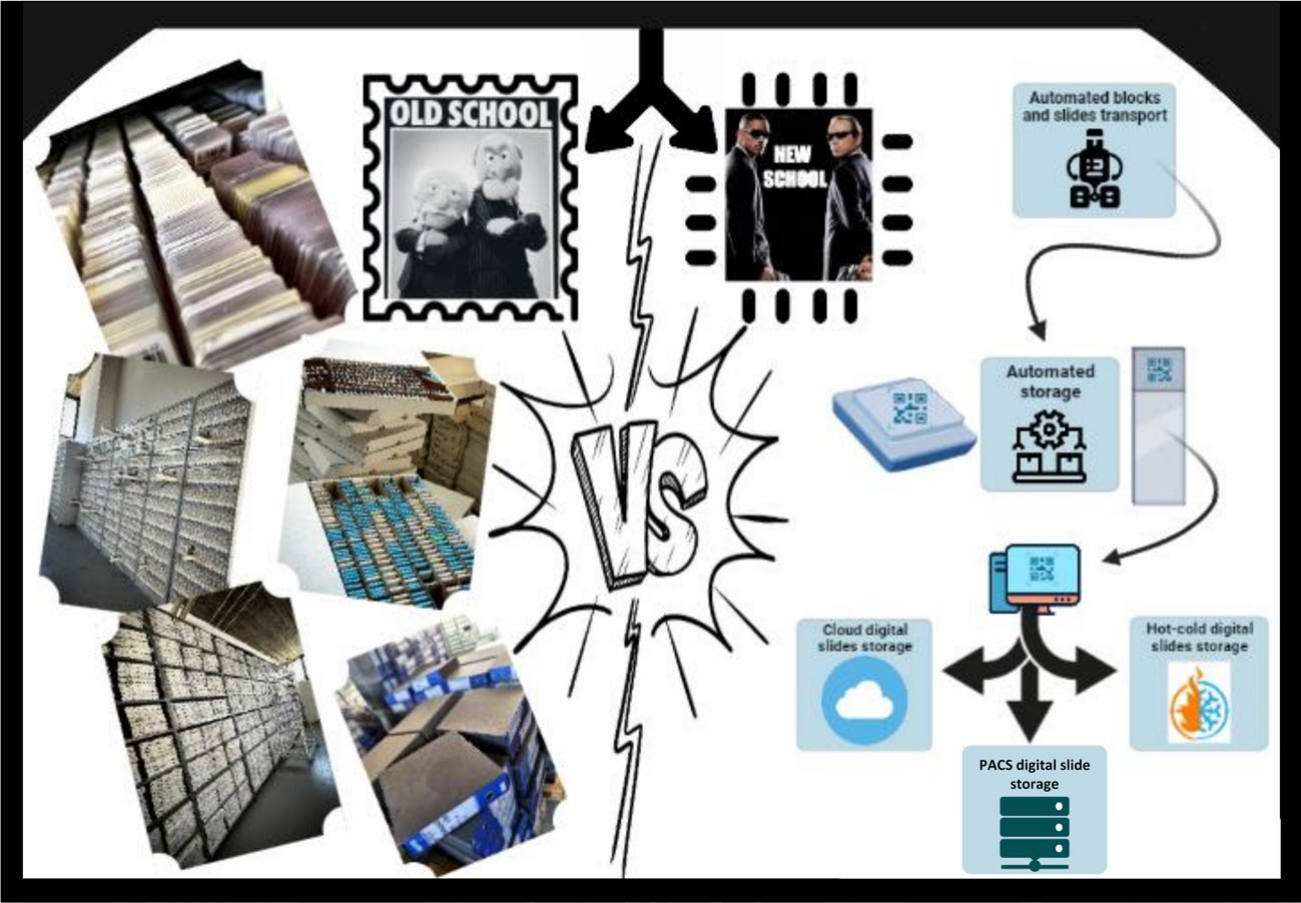
Shifting paradigms in storage and archiving

The application fields of automation in the analytical phase of pathology include storage and biobanking of tissues and specimens and digital imaging archiving (Fig. 3 and Table 3).

**Table 3:** Benefits of automation in the post-analytical phase: storage and archiving

Storage and biobanking of tissue and specimens	Digital imaging archiving
Proper preservation methods are vital for tissue and surgical specimens Preservation can involve freezing, refrigeration, or formalin fixation Automated storage solutions ensure traceability and efficiency *SmartCABINET and ClientCABINET (LOGIBIOTECH, Italy); Arkive BC™ (Menarini, Italy)*	Storage of WSIs and diagnostic data for future analysis, research, and decision-making Offers easy accessibility, faster retrieval, and resistance to deterioration VNAs store digital images in a format independent of vendors VNAs link digital pathology data with health care IT systems SNOMED CT vocabulary is crucial for organized data analysis in digital archives

*WSI*, whole slide images; *VNA*, vendor-neutral archives. Examples of products available on the market are presented in italics

### Storage and biobanking of tissue and specimens

Tissue and surgical specimens should be kept in a way that protects their integrity and averts deterioration or contamination. Depending on the specimen type and the intended purpose, this can entail freezing, refrigeration, or formalin fixation [44].

Professional associations such as the Joint Commission on Accreditation of Health Care Organizations (USA) and the College of American Pathologists (USA) advise that tissue blocks and slides be kept for a long enough time to ensure that the patient is treated properly. The UK’s Royal College of Pathologists advises keeping blocks for life and histology slides and smears for 10 years [45].

Different companies have created automated histology cassette storage and management solutions to improve traceability, speed up archiving and retrieval, protect sensitive patient tissue and biopsy blocks, and cut down on sorting and storage time [46].

To preserve lab security, increase productivity, and optimize procedures, built-in automation must be integrated. Reliable laboratory information system (LIS) integration and user-friendly software on board boost performance and lower errors. Complete traceability and straightforward cassette retrieval are also necessary for maintaining an efficient operation. Additionally, a safe, secure storage facility that is constantly watched over is needed to ensure the preservation, integrity, and quality of samples [47].

To make sure that each task is managed and monitored in the right and timely manner, many solutions have been put forth by vendors, such as Arkive BC™ (Menarini Diagnostics, Florence, Italy), which may be easily interfaced with middleware or included into the LIS as needed. Authorized technicians may comprehensively oversee all areas of the operations thanks to the user-friendly interface of the onboard software.

For histology/cytology slides, the same manufacturer has produced a product named Arkive SL™, a device for loading slides to be archived and the retrieval of archived slides. The SLTrack tool automatically records a full audit trail of each slide and SL item in the lab, enabling users to trace, identify, and retrieve samples quickly and efficiently. Additional units are added to build a modular system, which increases overall capacity without limiting the number of slides that can be retained for short-, medium-, and long-term storage requirements.

SmartCABINET and ClientCABINET (LOGIBIOTECH, Alseno, Italy) are smart units for the automated and traceable storage of cytohistological samples. They are an intelligent automated system which can help operators perform all phases of the storage and retrieval of cytohistological samples. Thanks to the software installed, “pick-to-light” technology, Wi-Fi connection, and the use of samAPP, each storage and retrieval operation is carried out in a secure, traceable, and recorded manner. They are modular and flexible, as numerous identical units can be added to each SmartCABINET, and each of these can in turn command an infinite number of ClientCABINETs to adapt to any storage volume requirement and space available.

### Digital imaging archiving

In pathology, digital image archiving describes the procedure of keeping WSIs and related diagnostic data for analysis and future use. The benefit of digital imaging archiving is that the data is always accessible and independent of traditional archives [48].

A digital archive makes information easier to find and faster to retrieve, resistant to deterioration, able to view earlier case comments, and simple to share with coworkers [49].

The methodology, high-volume scanning, and particularly the enormous storage capacity required, as well as the associated costs, provide unique problems when developing a fully digitized slide library [50].

Medical management systems that store and handle digital images and data in a vendor-neutral format are called vendor-neutral archives (VNAs). Regardless of vendor or manufacturer, a VNA may save WSIs and associated data from digital pathology systems and link with other health care IT systems like EHR and LIS. VNAs can store images on dedicated hardware or link to third-party systems’ images. Central VNAs enable image backup, disaster recovery, business continuity, and interoperability with external organizations and health information exchanges. When new imaging technologies like radiology PACS are introduced, they can reduce picture data migration. Federated VNA may be cheaper and faster to install due to their lower hardware infrastructure requirements, but they may have more trouble connecting to multiple image sources [51].

Digital imaging archiving enables pathologists to analyze enormous amounts of data and find patterns and trends for research and therapeutic decision-making. Systematized Nomenclature of Medicine Clinical Terms (SNOMED CT) is crucial. With over 350,000 ideas and a million relationships, SNOMED CT is the most comprehensive, multilingual clinical health care vocabulary in the world [52].

SNOMED topography (SMOMED T) and SNOMED morphology (M) codes could be used to choose cases from a digital archive, which could help minimize the archive’s size and cost while preserving the benefits of quick and easy retrieval of WSIs [49].

## Conclusions and future directions

Automation in surgical pathology has demonstrated immense potential for enhancing the accuracy, efficiency, and overall quality of patient care. Through the integration of advanced technologies such as robotics, AI, and machine learning, pathology laboratories can reduce human error, streamline workflows, and expedite the diagnostic process. As these innovations continue to evolve, it is essential for the medical community to embrace and adapt to these changes while addressing any ethical and legal concerns that may arise. The future of surgical pathology is undeniably intertwined with the advancements in automation, paving the way for more accurate diagnoses, improved patient outcomes, and a more profound understanding of diseases.

## Disclaimer

The cited platforms and vendors are meant to serve as examples stemming from the authors’ knowledge and experience. Such examples are not intended as endorsements and might not accurately represent the latest technological advancements.

## Data Availability

The authors confirm that the data supporting the findings of this study are available within the article.
